# Roles of Interleukin-9 in the Growth and Cholecystokinin-Induced Intracellular Calcium Signaling of Cultured Interstitial Cells of Cajal

**DOI:** 10.1371/journal.pone.0095898

**Published:** 2014-04-22

**Authors:** Yaoyao Gong, Lei Huang, Wenfang Cheng, Xueliang Li, Jia Lu, Lin Lin, Xinmin Si

**Affiliations:** 1 Department of Gastroenterology, The First Affiliated Hospital of Nanjing Medical University, Nanjing, China; 2 Department of Pediatric Surgery, Nanjing Children’s Hospital Affiliated to Nanjing Medical University, Nanjing, China; University of Nevada School of Medicine, United States of America

## Abstract

Interstitial cells of Cajal (ICC) are pacemaker cells in the gastrointestinal (GI) tract and loss of ICC is associated with many GI motility disorders. Previous studies have shown that ICC have the capacity to regenerate or restore, and several growth factors are critical to their growth, maintenance or regeneration. The present study aimed to investigate the roles of interleukin-9 (IL-9) in the growth, maintenance and pacemaker functions of cultured ICC. Here, we report that IL-9 promotes proliferation of ICC, and culturing ICC with IL-9 enhances cholecystokinin-8-induced Ca^2+^ transients, which is probably caused by facilitating maintenance of ICC functions under culture condition. We also show co-localizations of cholecystokinin-1 receptor and IL-9 receptor with c-kit by double-immunohistochemical labeling. In conclusion, IL-9 can promote ICC growth and help maintain ICC functions; IL-9 probably performs its functions via IL-9 receptors on ICC.

## Introduction

INTERLEUKIN-9 (IL-9), which is dependent upon Type 2 helper T cells, is a multifunctional cytokine that functions as either a positive or a negative regulator of immune responses. Mast cells also produce IL-9, and in turn IL-9 contributes to the growth of mast cell progenitors [1], [2]. IL-9 acts together with stem cell factor (SCF) to promote mast cell expansion [3]. Interstitial cells of Cajal (ICC) are pacemaker cells responsible for initiating slow-wave activity in the gastrointestinal (GI) tract [4]. Previous reports have shown that pacemaker activities in ICC might be generated by Ca^2+^ oscillators that are dependent upon IP3 receptor (IP3R)-mediated Ca^2+^ release and mitochondria-mediate uptake [5], [6].

Increasing research reveals that a reduction in ICC population and damage to the ICC network may contribute to dysmotility in patients with inflammatory bowel disease [7], [8]. ICC can be lost rapidly under certain circumstances; however, morphological studies have shown that ICC have the capacity to regenerate or restore [9], [10]. It is widely accepted that SCF, insulin-like growth factor-I (IGF-1) and insulin are critical to the development and functional maintenance of ICC [11], [12], [13]. In addition to these growth factors, research from Dr. Huizinga has revealed that IL-9 has a proliferative effect on ICC inside tissue explants and mast cells make membrane-to-membrane contact with injured ICC and exhibit piecemeal degranulation at the ultrastructural level [14], [15]. This data suggested that IL-9 secreted by mast cells may promote growth and repair of ICC, indicating the possibility that other kinds of cell factors may enhance ICC proliferation and restoration in addition to growth factors. However, ICC and mast cells are the only c-kit positive cells in the gut muscle and IL-9 enhances mast cell expansion together with SCF [3]. Therefore, it would be useful to determine whether IL-9 has a direct effect on ICC development or performs its function via SCF. Considering that SCF released by smooth muscle cells may affect the function of IL-9 in cultured tissue explants, the present study sought to examine the effects of IL-9 on the growth of cultured ICC. In addition, the role of IL-9 in the pacemaker activities of ICC is still unknown; this study also investigated the effect of IL-9 on intracellular Ca^2+^ concentration ([Ca^2+^]i) in ICC. Surprisingly, IL-9 exhibited no effect on [Ca^2+^]i. Whereas, the addition of IL-9 in ICC culture enhanced the cholecystokinin-8 (CCK-8)-evoked Ca^2+^response. Moreover, we observed the co-localizations of IL-9 receptor (IL-9R) and CCK_1_ receptor (CCK_1_R) with c-kit immunoreactivities in murine gastric antral tissues.

## Materials and Methods

### Ethical Approval

The animals used in the present study were treated ethically. All procedures were approved by Institutional Animal Care and Use Committee at Nanjing Medical University.

### Animals and Tissue Preparation

Balb/c mice (6–7 weeks) of either sex were purchased from the animal center of Nanjing Medical University. The animals were anesthetized by isoflurane inhalation and sacrificed by cervical dislocation. Stomachs were removed from the mice and the antrum was dissected out for subsequent use. The mucosa was removed by peeling. In Sylgard dishes filled with Krebs solution, the tissues were washed three times and then cut into approximately 0.5 cm segments.

### Preparation of Cells and ICC Purification by Fluorescence-Activated Cell Sorting

The tissue segments were labeled with the ICC marker c-kit conjugated with fluorescent dye PE-Cy7 (PC7, 13 µg/ml) for 3 h at 4°C. After labeling, the tissues were incubated at 37°C for 30 min with an enzyme solution containing collagenase 1.3 mg/ml, trypsin inhibitor 2 mg/ml and ATP 0.27 mg/ml. An equal volume Medium M199 (no phenol red, Gibco, Carlsbad, CA, USA) containing 10% fetal bovine serum (Hyclone, South Logan, Utah, USA) was added to stop digestion and cells were collected by pouring the suspension through a 200-mesh sieve.

The purification of ICC by fluorescence-activated cell sorting (FACS) was performed as described by Ordog T et al [11], [16]. Briefly, single-cell suspensions were incubated with the following antibodies: labeling of ICC was reinforced with PC7-c-kit (clone: ACK2, 0.5 µg/10^7^ cells in 100 µl M199, Biolegend, San Diego, CA, USA); mast cells and other leukocytes were identified using PC5-CD45 (0.25 µg/10^7^ cells in 100 µl M199, eBioscience, San Diego, CA, USA) antibody. In addition, cells were incubated with PC5-F4/80 (0.25 µg/10^7^ cells in 100 µl M199, eBioscience) and PC5-CD11b (0.5 µg/10^7^ cells in 100 µl M199, eBioscience) antibodies to label macrophages. We also employed PE-CD34 (0.4 µg/10^7^ cells in 100 µl M199, BD Biosciences, Franklin Lakes, NJ, USA) to identify ICC precursors. Cells were sorted on a BD FACSAria II instrument. Fluorescence-minus-one (FMO) controls were used to identify gating boundaries.

### Cell Culture and Counting Use a Light Microscope

Purified ICC were evenly divided into 5 groups (control, IL-9 0.001 µg/ml, IL-9 0.01 µg/ml, IL-9 0.1 µg/ml and IL-9 1 µg/ml; IL-9, Peprotech, Rocky Hill, NJ, USA). Briefly, dispersed cells were plated onto 35 mm-diameter culture dishes coated with 2.5 mg/ml rat tail collagen (Hangzhou Shengyou Biotechnology, Hangzhou, China) and cultured at 37°C in a 95%O_2_-5% CO_2_ incubator in M199 supplemented with 1% penicillin-streptomycin solution (Hyclone), 15% fetal bovine serum and different concentrations of IL-9. Following culturing for 6 days, ICC were counted in ten consecutive high-power fields (400×,/HP). The data was expressed as the mean number of cells per high-power field. After counting, total RNA was extracted and quantitative RT-PCR was performed.

### Quantitative RT-PCR

Quantitative RT-PCR was used to assess the specificity of the ICC sorting and the effect of IL-9 on c-kit mRNA expression. Total RNA was isolated using the Trizol reagent following the manufacturer’s instructions. RNA was quantified using the RNA 6000 Nano assay (Agilent Technologies, Santa Clara, CA, USA). The cDNA reverse transcription product was amplified with specific primers (see below) by PCR, using the following amplification profile: 95°C for 10 min, followed by 50 cycles of 95°C for 15 s and 60°C for 1 min. Real-time quantitative PCR was performed using SYBR Green chemistry on a StepOne Real-Time PCR system (Applied Biosystems, Carlsbad, CA, USA). Transcriptional quantification was obtained relative to the GAPDH standard curve. The following primers were used: c-kit (Y00864): sense, 5′-CGCCTGCCGAAATGTATGACG-3′; antisense, 5′- GGTTCTCTGGGTTGGGGTTGC-3′; mast cell tryptase (MCT; M57626): sense, 5′- GTGGGACCGCACATCAAAAG-3′; antisense, 5′- TCAAGCTCCAGCAGGGCAAC-3′; smooth muscle myosin heavy chain (MyHC; NM_013607): sense, 5′-GGTGGAGGATGAGCGCAAGATGGCA-3′; antisense, 5′-TTCCTGTGGGGGGGGCCCTCTGAGT-3′; CD68 (NM_009853; a pan-macrophage marker): sense, 5′-ATAGCCCAAGGAACAGAGGAAGACT-3′; antisense, 5′-GTTATGAGTGACAGTTGTGGGTCCG-3′; GAPDH (NM_008084): sense, 5′-AGTATGACTCCACTCACGGCAA-3′; antisense, 5′-TCTCGCTCCTGGAAGATGGT-3′.

### Measurement of Intracellular Ca^2+^ Concentration

Changes in Ca^2+^ activity were monitored using Fluo-3/AM (Invitrogen, Carlsbad, CA, USA). The cultured ICC grown on glass-bottom dishes (NEST Biotechnology Co., Wuxi, China) were rinsed twice with PBS, and then incubated in M199 containing 5 µM Fluo-3/AM in a 95%O_2_-5%CO_2_ incubator for 40 min. Following two more rinses, the dishes were scanned every 50 ms with a confocal laser scanning microscope (LSM710, Zeiss, Germany). Fluorescence was excited at a wavelength of 488 nm, and emitted light was observed at 515 nm. The variations of Ca^2+^ fluorescence emission intensity were expressed as F/F0, where F0 was the intensity of the basal fluorescence level before addition of chemicals. CCK-8 (Sigma Aldrich, St. Louis, MO, USA) was employed as a stimulant to evoke Ca^2+^ transients, and carbachol (CCh, Sigma Aldrich) was used as a positive control.

### Immunostaining of Cultured ICC

Cultured ICC were fixed with acetone (4°C, 8 min). Following fixation, preparations were washed for 5 minutes(×3) in phosphate buffered saline (PBS; 0.01M, pH 7.4) and then incubated in blocking buffer (10% goat serum and 0.1% Triton in PBS) for 30 minutes to reduce nonspecific antibody binding and increase penetration of the antibodies. Then, cells were incubated overnight at 4°C with a mixture of primary antibodies. The primary antibodies included the following: rabbit anti-ANO1 polyclonal antibody (2 µg/ml, Abcam, Cambridge, UK); rabbit anti-cholecystokinin-1 receptor (CCK_1_R) polyclonal antibody (10 µg/ml, Bioworld, St. Louis Park, MN, USA); rat anti-c-kit monoclonal antibody (5 µg/ml, Abcam); and rabbit anti-IL-9R polyclonal antibody (2 µg/ml, Santa Cruz, Santa Cruz, CA, USA). Immunoreactivity was detected by incubation for one hour using a mixture of secondary antibodies. Secondary antibodies used were as follows: Alexa Fluor (AF) 555 goat-anti-rabbit antibody (4 µg/ml, Invitrogen), AF 488 goat anti-rabbit antibody (4 µg/ml, Invitrogen) and AF594 rabbit-anti-rat antibody (4 µg/ml, Invitrogen). Nuclei were stained with DAPI. For negative controls, cells were treated in the same way omitting the primary antibody. Reactions were examined with the confocal scanning laser microscope.

### Double-Immunohistochemical Staining on the Murine Gastric Antral Tissue

Murine gastric antral tissues were routinely processed and embedded in paraffin. Serial sections were cut and mounted on slides. The sections were deparaffinized and then incubated in a citrate buffer solution (pH 6.0) for 15 minutes in a microwave oven (96°C), followed by cooling at room temperature for 15 minutes. For whole-mount preparations, the gastric antral tissues was fixed with cold acetone (4°C, 1 hour) and opened along the lesser curvature of the stomach, then the mucosa and submucosa were removed by sharp dissection. The sections or whole-mount tissues were treated with 0.3% Triton X-100 to increase penetration of the antibodies and 10% goat serum to reduce non-specific binding. For the simultaneous visualization of two antigens, an indirect double immunofluorescence procedure was employed. The sections or whole-mount preparations were incubated overnight at 4°C with a mixture of primary antibodies. The primary antibodies used were the antibodies mentioned above. After washing with PBS, the slides or whole-mount tissues were incubated for 1 h at room temperature with a mixture of secondary antibodies. Secondary antibodies used were the antibodies used in the immunostaining of cultured cells. Negative controls were treated in the same way but omitted the primary antibodies. The whole-mount tissues and tissue sections were observed with the confocal laser scanning microscope or a fluorescence microscope.

### Statistical Analyses

Data are expressed as mean ± standard error of the mean (SEM). Differences in the data were evaluated by ANOVA or Student’s t test. Zeiss Zen 9.0 was used to analyze the calcium intensity data and GraphPad Prism 5.0 for charting. SPSS 11.0 was used for statistical analyses. Differences between control and test values were considered significant when *P*<0.05.

## Results

### Purification of ICC by Fluorescence-Activated Cell Sorting

**Figure 1:**
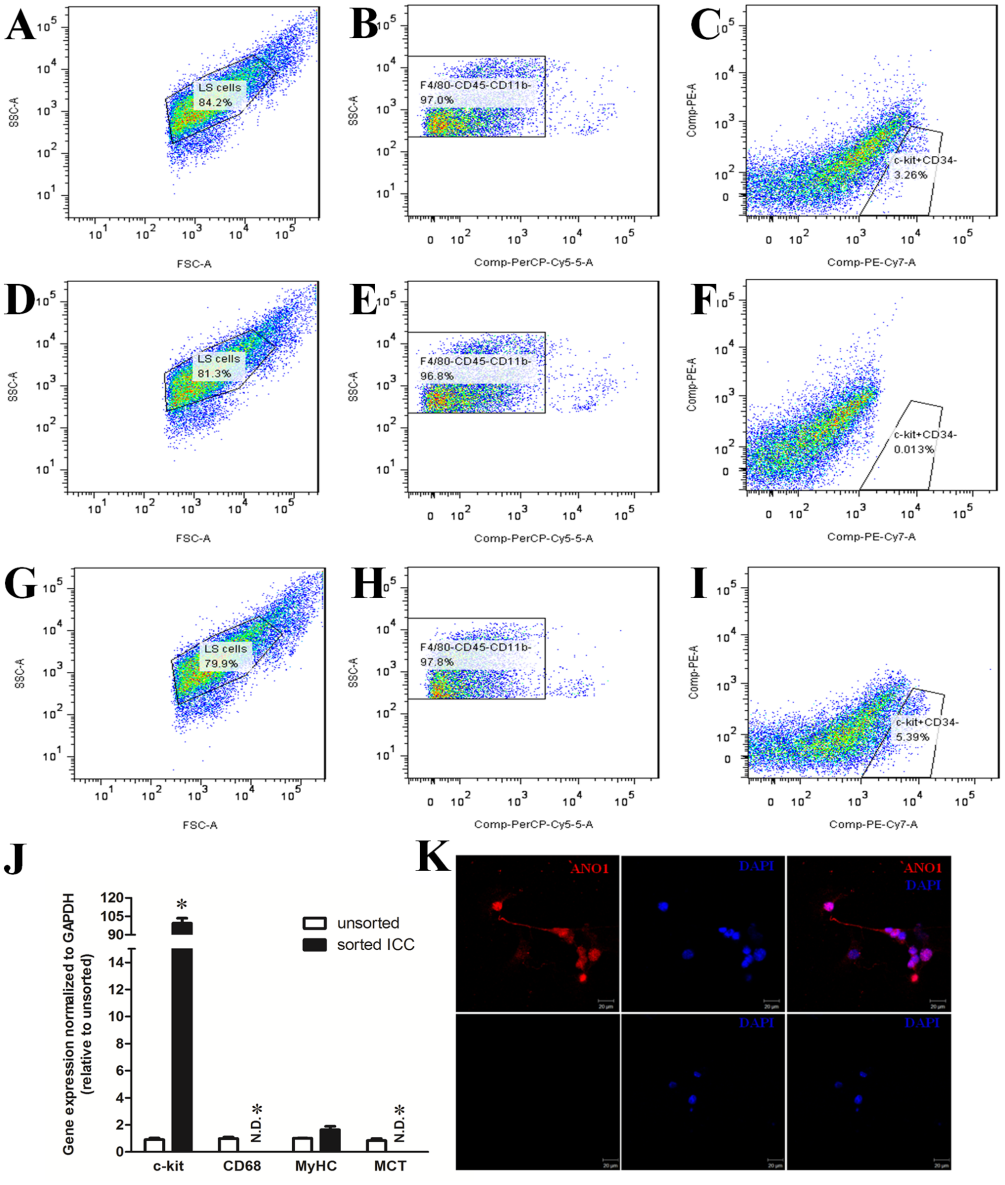
Sorting of ICC by FACS and identification of ICC. (A, D, G) Gates used for selecting cells with light scatter properties of live cells (LS cells). (B, E, H) Gates used for selecting cells that did not express macrophage markers (F4/80, CD11b) and pan leukocyte marker CD45 (PC5^−^ cells). (C, F, I) Gates used for separating ICC progenitors (c-kit^+^CD34^+^ cells) and mature ICC (c-kit^+^CD34^−^ cells). (A–C) Samples labeled with all markers. (D–F) FMO control without the c-kit antibody. (G–I) FMO control without the CD34 antibody. (J) Quantitative RT-PCR analysis of gene expression in the sorted ICC. Gene expression was normalized to GAPDH mRNA levels and expressed relative to the unsorted cells. Note the significant enrichment of ICC in the sorted ICC group. The expression of CD68 (a macrophage marker) and MCT (a mast cell marker) were not detected. However, MyHC (a smooth muscle cell marker) was observed but did not increase after sorting. (K) Immunohistochemical staining of cultured ICC using an antibody against ANO1 (red); nuclei were stained with DAPI (blue); negative control were treated in the same way but omitted the primary antibody.

First, forward scatter (FSC)-width and side scatter (SSC)-height gates were set to select cells with light scatter properties of live cells (LS cells; Fig. 1A, D, G). Second, the F4/80^−^CD45^−^CD11b^−^ cell gates were set to exclude macrophages, mast cells and other leukocytes (Fig. 1B, E, H). Next, mature ICC (c-kit^+^CD34^−^ cells; Fig. 1C) were separated from ICC precursors (c-kit^+^CD34^+^ cells). Finally, those mature ICC were harvested and cultured. Fluorescence-minus-one (FMO) controls (Fig. 1D–I) were used to help identify gating boundaries.

To examine the purity of ICC sorting, we used quantitative PCR with both unsorted cells and sorted ICC. The results (Fig. 1J) indicated a 99.4±4.1− fold (Student’s t test, *P*<0.0001) enrichment of c-kit expression in sorted ICC relative to unsorted cells. Smooth muscle myosin heavy chain (MyHC) expression was detected but did not increase in the sorted cells. CD68 (a macrophage marker) and mast cell tryptase (MCT) mRNA were not detected in the sorted group.

### Identification of ICC by ANO1 Immunofluorescence

As shown in Fig. 1K, cultured ICC identified by ANO1 immunofluorescence had distinctive shapes such as spindle, triangular or stellar-like with two to five long processes.

### Effect of IL-9 on ICC Proliferation

**Figure 2:**
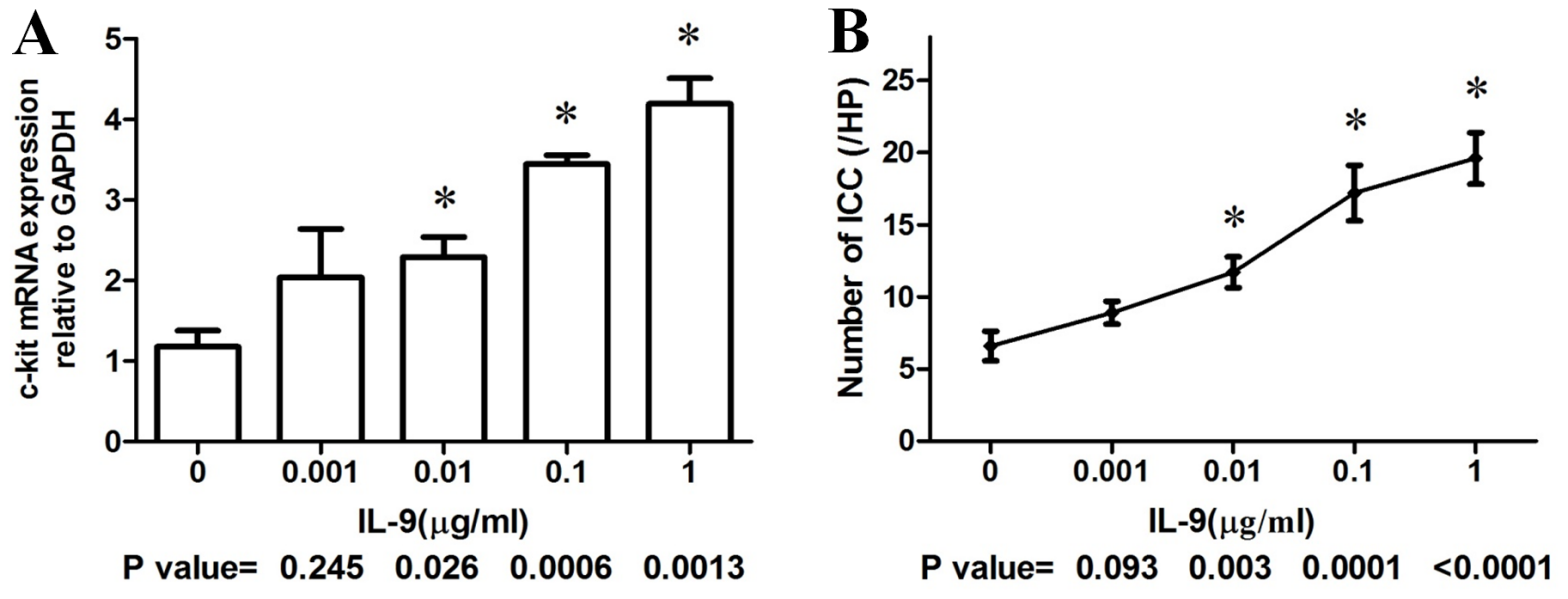
Effects of IL-9 on the ICC development *in vitro*. (A) Quantitative RT-PCR analysis of c-kit expression in cultured ICC. Culturing ICC with IL-9 (0.001∼1 µg/ml) for 6 days induced a dose-dependent increase in c-kit expression. (B) Cell counting results after culturing with or without IL-9 for 6 days. The number of ICC was calculated as per high-power field (/HP). Data were obtained from at least four different culture dishes. *P*-values were obtained by comparing each group with the control (IL-9 0 µg/ml) using One-way ANOVA.

To examine the effect of IL-9 on ICC development, ICC were cultured for 6 days with or without IL-9. The concentrations of IL-9 ranged from 0.001 µg/ml to 1 µg/ml. In the presence of 0.001 µg/ml IL-9, ICC did not show marked proliferation; whereas, addition of 0.01, 0.1 or 1 µg/ml IL-9 all promoted ICC expansion, as shown in Fig. 2B (differences in the data were evaluated by One-way ANOVA). The quantitative PCR results coincided with the cell counting results. IL-9 caused a dose-dependent increase in c-kit mRNA expression (Fig. 2A).

### IL-9 Enhanced CCK-8 or CCh-Induced Ca^2+^ Transients in ICC

**Figure 3:**
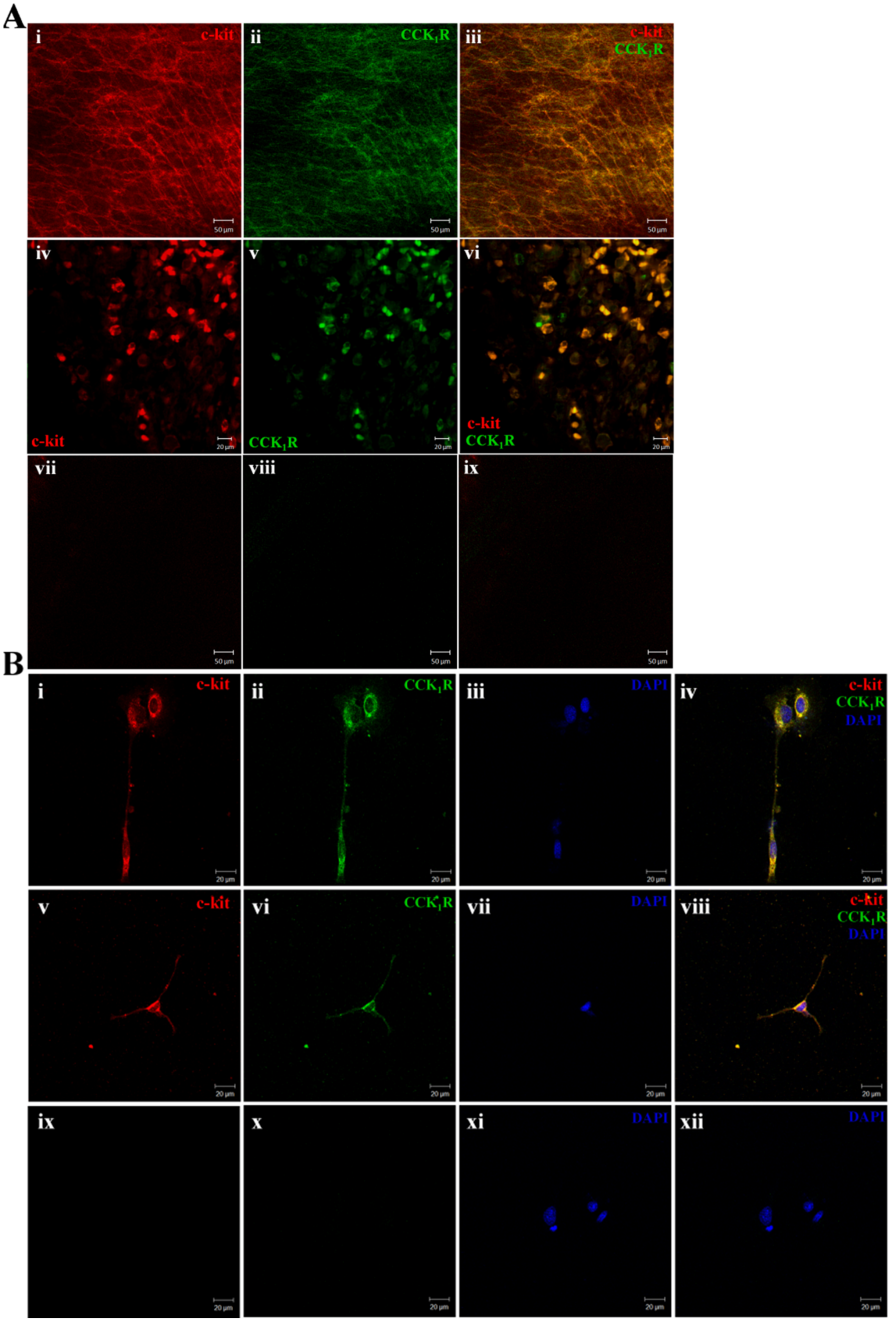
Detection of CCK_1_R on murine gastric antral ICC by double-immunohistochemical labeling. (Ai-iii) Double immunofluorescent labeling of c-kit (red) and CCK_1_R (green) in the tunica muscularis of the gastric antrum (whole-mount preparations). Most, but not all, CCK_1_R-immunopositive cells were c-kit-immunopositive. (Aiv-vi) Double immunohistochemistry of c-kit and CCK_1_R in the muscular layer of the gastric antrum (transverse section). (Avii-ix) Negative control omitting the primary antibody. (B) Double immunofluorescent labeling of c-kit and CCK_1_R on cultured ICC. (Bi-iv) An example of ICC-IM-like cells that have bipolar shape. (Bv-viii) An example of ICC-MY-like cells that have triangular shape. (Bix-xii) Negative control omitting the primary antibody. Nuclei were stained with DAPI (blue).

As intracellular Ca^2+^ oscillations in ICC are considered the primary mechanism for the pacemaker activity in GI motility, we examined the effect of IL-9 on [Ca^2+^]i in ICC. IL-9 at concentrations ranging from 0.001 to 0.1 µg/ml had no effect on [Ca^2+^]i in ICC (not shown). However, we found that culturing ICC with IL-9 could improve the effect of CCK-8 on [Ca^2+^]i. We previously reported that CCK-8 could evoke Ca^2+^ transients in ICC [17]. Furthermore, we found that c-kit positive cells in the murine gastric antrum co-localized with the CCK_1_ receptor (CCK_1_R) (Fig. 3A), and the cultured ICC expressed CCK_1_R as well as c-kit (Fig. 3B). On the basis of our previous study, we concluded that CCK-8 could reinforce the pacemaker activities of ICC via CCK_1_R.

**Figure 4:**
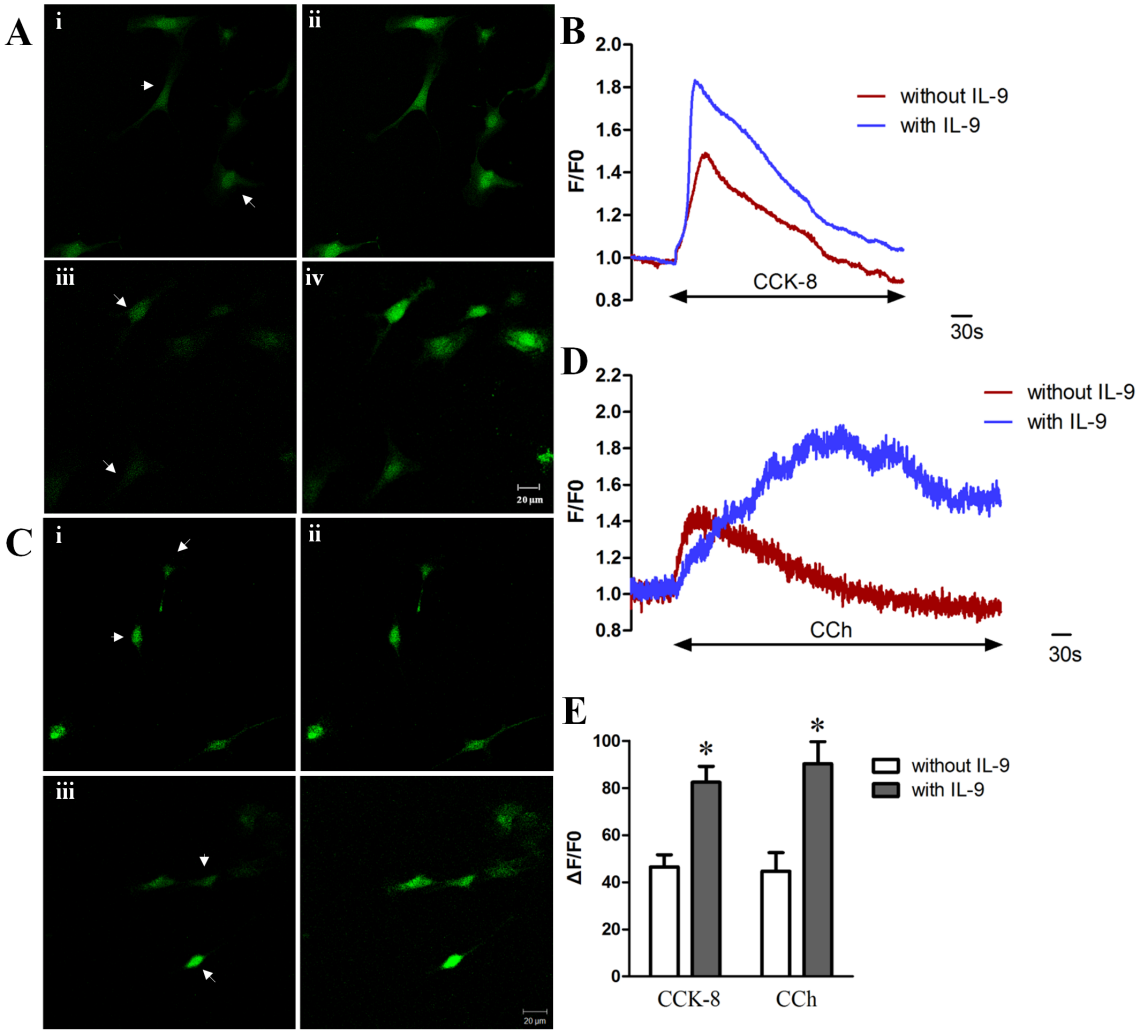
Effects of CCK-8 or CCh on [Ca^2+^]i in ICC cultured with or without IL-9. (Ai) The basal fluorescent image of [Ca^2+^]i in ICC cultured without IL-9. (Aii) The peak point image in the presence of CCK-8 (10^−7 ^mol/L). (Aiii) The basal fluorescent image of [Ca^2+^]i in ICC cultured with 0.1 µg/ml IL-9. Culturing with IL-9 did not elevate the basal [Ca^2+^]i of ICC. (Aiv) The peak fluorescent image after addition of CCK-8 (10^−7 ^mol/L). Responses of ICC to CCK-8 were markedly increased. (B) Average effect of CCK-8 on [Ca^2+^]i over time (n = 8). Culturing ICC with IL-9 for 6 days elevated the response of [Ca^2+^]i to CCK-8, but did not increase the basal [Ca^2+^]i. (Ci)The basal fluorescent image of [Ca^2+^]i in ICC cultured without IL-9. (Cii) The peak point image in the presence of CCh (10^−5 ^mol/L). (Ciii) The basal fluorescent image of [Ca^2+^]i in ICC cultured with 0.1 µg/ml IL-9. (Civ) The peak fluorescent image after addition of CCh (10^−5 ^mol/L). (D) Average effect of CCh on [Ca^2+^]i over time (n = 6). (E) Effects of CCK-8 or CCh on ICC [Ca^2+^]i were estimated as percentage of ΔF/F0, where F0 was derived from the averaged intensity of the first 10–30 frames minus the background. ΔF is the peak fluorescent intensity stimulated by CCK-8 or CCh minus F0. *P<0.05 compared with ICC cultured without IL-9 using Student’s t test. Arrows point to the cells which were selected for the Ca^2+^ imaging.

When we used ICC that had been cultured with 0.1 µg/ml IL-9 for 6 days, we found that the responses of ICC to CCK-8 were improved (Fig. 4A, B, E). In ICC cultured without IL-9, CCK-8 (100 nmol/L) increased the mean [Ca^2+^]i by 46.42% ±5.26% (Fig. 4E). Whereas, CCK-8 elevated [Ca^2+^]i by 82.45% ±6.73% in ICC cultured with IL-9 (Fig. 4E). In the positive control group, 10^−5^ mol/L CCh-induced Ca^2+^ response could also be reinforced by IL-9 incubation (90.24% ±9.42% vs 44.68% ±7.93%, Fig. 4C, D, E).

### Gastric Antral ICC Express IL-9R

**Figure 5:**
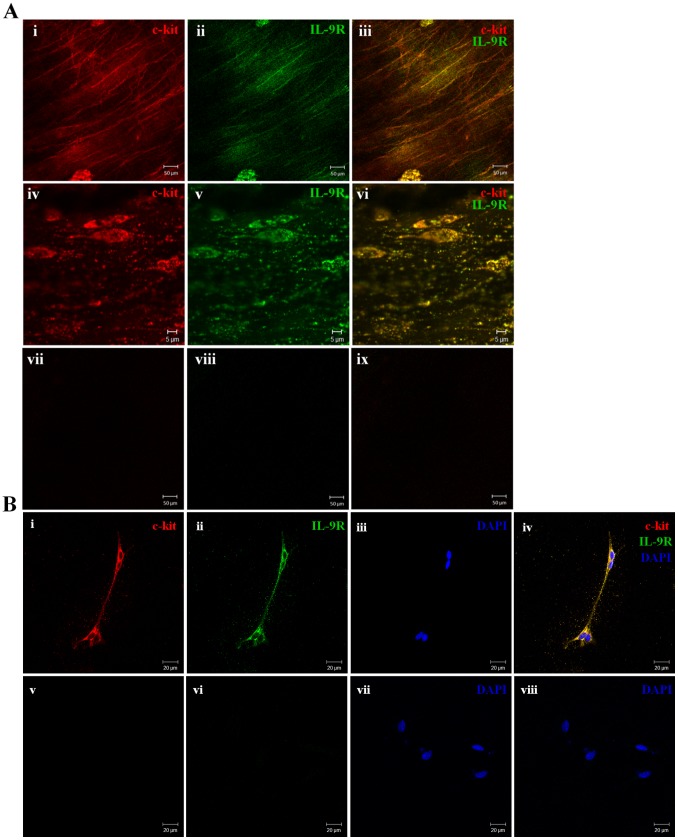
Expression of the IL-9R was shown by immunohistochemistry on murine gastric antral ICC. (Ai-iii) Double immunofluorescent labeling of c-kit (red) and IL-9R(green) in the tunica muscularis of the gastric antrum (whole-mount preparation). (Aiv-vi) Double immunohistochemistry of c-kit and IL-9R in the circular muscle layer (sectioned antral tissue). (Avii-ix) Negative control. (Bi-iv) Double immunofluorescent labeling of c-kit and IL-9R on cultured ICC. Note that all the c-kit-immunopositive cells showed IL-9R immunoreactivity. (Bv-viii) Negative control.

We then detected whether the IL-9R was expressed by murine gastric ICC. Double staining with anti-c-kit and anti-IL-9R antibodies revealed that all c-kit positive cells displayed IL-9R immunoreactivity (Fig. 5A). Although c-kit is expressed by both ICC and mast cells, we know that mast cells only represent a small fraction of c-kit positive cells. C-kit positive cells (both in whole mount and sectioned antral tissues) and cultured ICC showed IL-9R immunoreactivity. (Fig. 5B); therefore, we concluded that ICC, as well as mast cells, express IL-9R.

## Discussion

It has been well documented that ICC play critical roles in GI motility and the loss of ICC is associated with many GI motility disorders. Therefore, it is important to identify those factors that improve ICC growth, maintenance and regeneration. The work of Ordog et al., suggests that (1) maintenance and survival of ICC requires insulin-like growth factor-I (IGF-1)/insulin and membrane-bound SCF; (2) IGF-1 promotes differentiation of ICC precursors to mature ICC; and (3) soluble SCF expands ICC progenitors [11], [13], [18]. The fact that IL-9 promotes the growth and maintenance of ICC inside tissue explants found by Dr. Huizinga’s research team [14], [15] illuminates the potential value of IL-9 as a supplement in ICC culture and a therapeutic factor in ICC restoration.

IL-9 is considered a growth-promoting agent for several cell types, such as CD4^+^ T cells, B cells, mast cells and hematopoietic progenitor cells [1]. Both direct and indirect effects are involved in those functions. In the present study, IL-9 was employed as a supplement in ICC culture, and it successfully increased c-kit mRNA levels and the number of ICC. SCF-secreting cells (e.g. fibroblasts and smooth muscle cells) were mainly excluded during cell sorting; therefore, it could be concluded that IL-9 enhances the growth of ICC directly. Furthermore, only mature ICC were gathered, suggesting that IL-9 promotes proliferation of mature ICC directly. IL-9 acting as an enhancer for the growth of mast cells is SCF-dependent; therefore, the mechanisms underlying proliferative activities of IL-9 on these two kinds of cells may be different [3].

IL-9R has two subunits: α-chain (IL-9Rα) and γ-chain which is shared by other cytokines, including IL-2, IL-4, and IL-7 [19], [20]. IL-9R is commonly expressed on T cell lines and effector T cells, and has also been found in human airway polymorphonuclear neutrophils, smooth muscle cells (in asthmatics but not in healthy individuals) and mast cells [1], [20], [21], [22]. In this study, IL-9R immunoreactivity was found in all c-kit^+^ cells, indicating the effects of IL-9 are probably mediated by the IL-9R on ICC. With respect to signal transduction, a previous study showed that phosphorylation of a tyrosine residue (Tyr^407^) in IL-9Rα and activation of three different STAT proteins is necessary for the distinct activities of IL-9, including proliferative responses [23]. Further studies are needed to investigate if this signal transduction also plays an important role in IL-9-induced ICC growth.

In Ca^2+^ signaling experiments, IL-9 did not increase [Ca^2+^]i in ICC, whereas the addition of IL-9 during culture improved CCK-8-evoked Ca^2+^ activity. In addition, double-immunohistochemical staining showed that ICC in the murine gastric antrum express CCK_1_R. We previously reported that CCK-8 could induce an apparent Ca^2+^ transient. Taken together, the two studies suggest that the biological effects of CCK-8 probably occur via CCK_1_R located on ICC and are dependent upon the release of IP3R-dependent intracellular Ca^2+^ release from the endoplasmic reticulum. In this study, CCh-evoked Ca^2+^ activity could also be reinforced by IL-9 incubation. Our data indicated that IL-9 probably facilitates maintenance of ICC function under culture condition, which promotes the responsiveness of ICC to CCK-8 or CCh. However, *in vivo* experiments should be done to confirm the effects of IL-9, which is a limitation of this study. On the other hand, the GI motor activity is dependent on the complex integration of ICC networks, nerves and smooth muscles. Therefore, measuring Ca^2+^ oscillations in tissues should be done to illustrate the effect of IL-9 on ICC pacemaker activities in the presence of the complete regulation system.

ICC exhibited spontaneous Ca^2+^ transients in many Ca^2+^ signaling studies, most of which were performed using tissues or short-term (less than 3 days) cultured cells [24], [25], [26], [27]. However, in this study, we did not detect spontaneous Ca^2+^ signals in cultured ICC. One possible reason is that the spontaneous Ca^2+^ signals weakened with the extension of culture time. This is consistent with studies of other cell types, such as cardiac myocytes. The systolic Ca^2+^ fluorescence intensity in cardiac myocytes decreased significantly along the culture time, which probably represents an adaptation to the culture conditions [28]. Moreover, some other studies observed little or no rhythmic spontaneous Ca^2+^ signal in ICC [29], [30]. Whether or not spontaneous Ca^2+^ transients were observed, increases in [Ca^2+^]i in ICC were elicited by exogenous stimulators.

ICC only represent a small fraction of cells in the GI tract; therefore, it is necessary to sort ICC before culture [16]. FACS increased the enrichment of ICC, but many of them were damaged during the sorting process and failed to grow. After repeating the experiments, cell survival was improved and the number of attached ICC increased. Nevertheless, it remains difficult to obtain sufficient protein from cultured ICC to perform western blotting.

Another limitation of this study is related to the ICC subpopulations. Previous studies have demonstrated that there are at least two subpopulations in the gastrointestinal tract: a population of ICC in the myenteric region (ICC-MY) and a second population of ICC distributed throughout the muscle layers (ICC-IM) [31]. ICC-MY are identified as multipolar cells while ICC-IM are bipolar cells. Whilst ICC-MY are regarded as the fundamentally important pacemaker cell type, ICC-IM are thought to serve an important function as intermediaries of motor neural input to the gastric musculature [32]. Further investigations into the effects of IL-9 on these two subpopulations should be carried out.

Taken together, the data obtained from our study suggest that: (1) IL-9 promotes proliferation of cultured ICC; (2) IL-9 reinforces the CCK-8 or CCh-induced Ca^2+^ transients in ICC; (3) IL-9 may facilitate maintenance of ICC function under culture condition; (4) IL-9 probably exerts its functions via IL-9R expressed by ICC.
